# De-escalated radiation for human papillomavirus virus-related oropharyngeal cancer: evolving paradigms and future strategies

**DOI:** 10.3389/fonc.2023.1175578

**Published:** 2023-07-27

**Authors:** Allen M. Chen

**Affiliations:** Department of Radiation Oncology, Chao Family Comprehensive Cancer Center, School of Medicine, University of California- Irvine, Irvine, CA, United States

**Keywords:** HPV, head and neck, cancer, radiation, squamous cell

## Abstract

The incidence of human papillomavirus (HPV)-associated oropharyngeal squamous cell carcinoma has increased dramatically in recent years reaching epidemic-like proportions. Data has emerged not only showing that these cancers are a unique entity with distinct molecular characteristics but that they also have a significantly improved prognosis as a result of their exquisite radiosensitivity compared to their HPV-negative counterparts. This, it has been increasingly suggested that these tumors can be targeted with de-escalated approaches using reduced doses of radiation. The overriding goal of de-escalation is to maintain the high cure and survival rates associated with traditional approaches while reducing the incidence of both short- and long-term toxicity. Although the exact reason for the improved radiosensitivity of HPV-positive oropharyngeal carcinoma is unclear, prospective studies have now been published demonstrating that de-escalated radiation can successfully maintain the high rates of cure and preserve quality of life for appropriately selected patients with this disease. However, these studies have been complicated by such factors as the relatively limited sample sizes, as well as the variability in treatment, inclusion criteria, and follow-up. As the data continues to mature on de-escalation, it is unquestionable that treatment paradigms for this disease will evolve. The ongoing quest to define a standard regimen comprises the subject of this review.

## Introduction

The incidence of human papillomavirus (HPV)-associated oropharyngeal squamous cell carcinoma has risen steadily in recent years reaching epidemic-like proportions. For many patients, radiation therapy is recommended as initial treatment given its longstanding track record and the excellent cure rates generally observed (1). Historically, this regimen, when used as primary treatment, has consisted of 7 weeks of daily radiation to relatively high doses, often combined with cisplatin chemotherapy. However, due to the anatomical volume of tissue requiring treatment, this regimen can be rigorous and difficult to tolerate with a significant proportion of patients developing long-term toxicity including dysphagia, xerostomia, neuropathy, and/or neck fibrosis (2). Unfortunately, these side effects can be severe, life-altering, and permanent. Indeed, the detrimental effect of treatment on quality of life, psychosocial health, and overall functional capacity has been well-established (3).

A plethora of clinical evidence has accumulated demonstrating that patients with HPV-positive oropharyngeal squamous cell carcinomas have an improved prognosis compared to their counterparts with HPV-negative disease (4–6). Furthermore, the recognition that HPV-positive oropharyngeal cancer responds exquisitely favorably to radiation, both in the pre-clinical and clinical settings, has prompted investigators to suggest that patients with these tumors are possibly over-treated and unnecessarily subjected to the toxicity of intensive chemoradiation with excessively high radiation doses. As a result, prospective trials have been conducted investigating the role of treatment de-escalation with the aim of reducing side effects, particularly those related to swallowing and salivary function, while maintaining the high rates of cure historically observed (7–12). Since patients with HPV-positive oropharyngeal cancer are often healthy, without medical comorbidities, and can potentially survive for decades after treatment, the focus on decreasing long-term complications and optimizing quality of life is particularly germane. For patients who are newly diagnosed with this disease, the focus on preserving function and maximizing well-being has taken on renewed importance. Indeed, the impetus for de-escalation for HPV-positive oropharyngeal carcinoma lies in discovering a new standard that preserves the precious balance between cure and quality of life to the fullest.

## Clinical data

**Figure 1:**
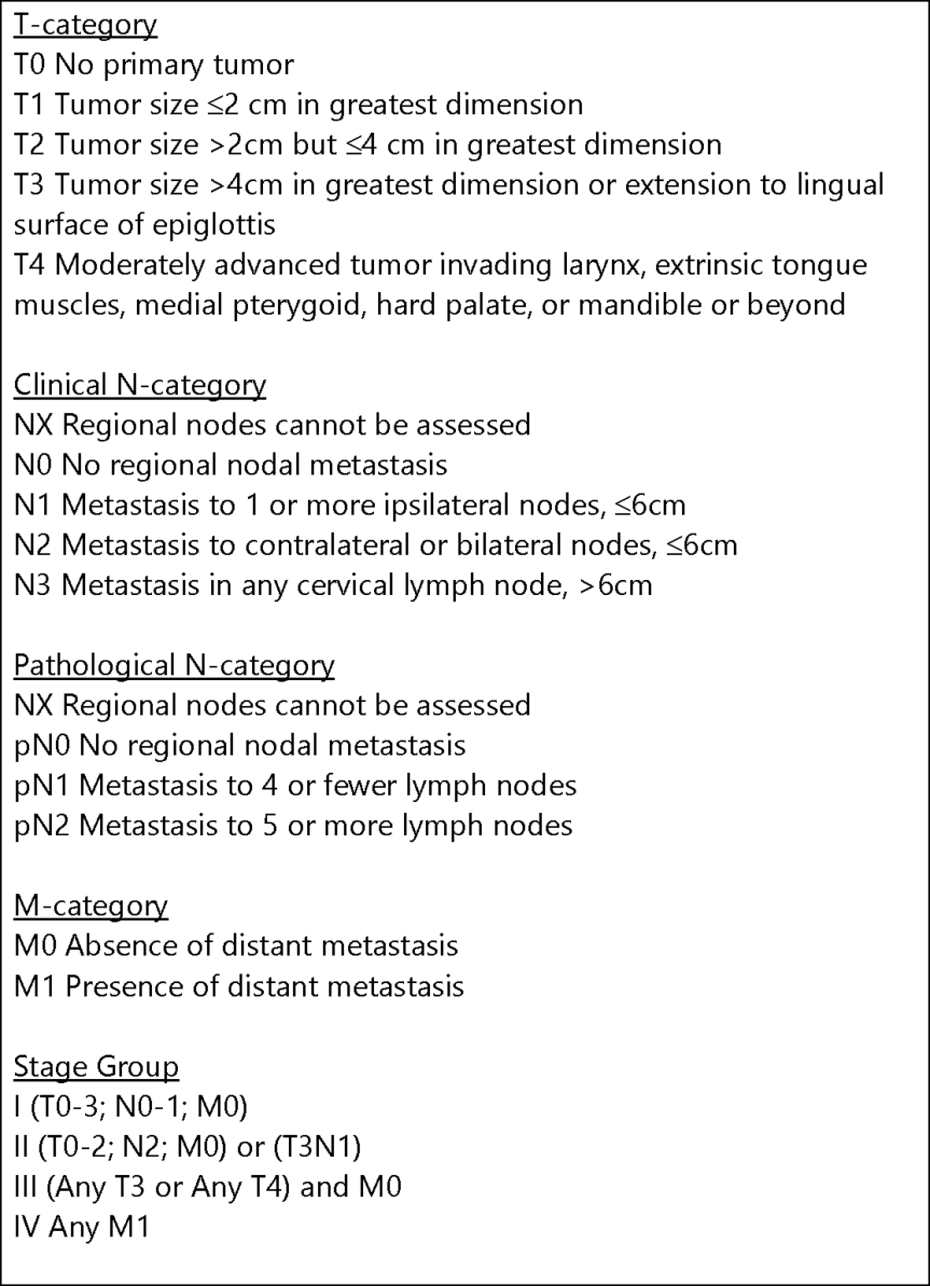
AJCC Staging System (Eighth Edition) for HPV-positive (p16-positive) squamous cell carcinoma of the oropharynx (13).

Clinico-pathologic biomarker investigations from clinical trials and retrospective studies have so convincingly confirmed HPV status as the single most important predictor of radiation response among oropharyngeal cancer patients that HPV staining (typically through the use of its surrogate, p16) is now standardly performed both in the community and in academic settings. Although HPV testing was initially conducted strictly for purposes of prognostication, its utility to assist with treatment decision-making has become increasingly apparent. Historic data initially published from the Radiation Therapy Oncology Group (RTOG) robustly demonstrated the prognostic importance of HPV. In this analysis, a wide difference was observed in the 3-year rates of local-regional control (86% vs. 65%) and overall survival (82% vs. 57%) between 433 patients with HPV-positive and HPV-negative phenotypes treated prospectively by cisplatin-based chemoradiation (5). Similarly, a subset analysis of 96 patients treated with an induction-concurrent chemoradiation regimen using a taxane-based regimen by the ECOG group showed that patients with HPV-positive tumors had significantly higher response rates (84% vs. 57%), overall survival (95% vs. 62%), and progression-free survival (86% vs. 53%) at 2-years (6). Given the strong link between HPV and radiation response, the American Joint Committee on Cancer (AJCC) created a new (Eighth) staging system in 2016 (**Figure 1**) specifically for patients diagnosed with HPV-positive oropharyngeal cancer to reflect its favorable prognosis compared to those with HPV-negative disease (13). Interestingly, many tumors that had been previously categorized as stage IV were significantly “down-staged” to stage II or even stage I cancers. This staging system has now been independently validated by numerous studies— overwhelmingly confirming the prognostic significance of HPV (14, 15). It is however important to recognize that the AJCC staging system, similar to most clinical trials, have considered p16-positivity to be equivalent to HPV-positivity. However, it is now established that patients with p16-positive/HPV-negative squamous cell oropharyngeal carcinomas do not have the same favorable prognosis as those with p16-positive/HPV-positive tumors—but rather one that is intermediate those of p16-positive/HPV-positive and p16-negative/HPV-negative cancer (16). As HPV-driven carcinomas are dependent on the permanent over-expression of the HPV E6 and E7 viral oncogene mRNAs, the presence of E6/E7 mRNA is considered to be the gold standard for identifying HPV-positive head and neck cancers (17). From a practical standpoint, mRNA testing is not done in practice because the results from p16 immunohistochemistry and *in situ* hybridization for HPV are generally considered reliable enough for clinical decision-making. It must be recognized, however, that concordance rates between tests can still be variable (17–21).

Furthermore, published data have also suggested that the favorable impact of HPV on prognosis is particularly strong for those patients deemed “never smokers” with several groups showing that the conferred benefit associated with HPV is attenuated for those with an increased smoking history (22). While controversy exists regarding how smoking and its intensity (as well as the impact of quitting) affects prognosis, it is generally accepted that an increased pack-year history and current smoking status are associated with worse outcome (22–25). **Table 1** illustrates the improved outcomes for HPV-positive oropharyngeal cancer from the radiation literature. Although the role of HPV in determining prognosis has been unequivocally established, questions persist on how to use this information in the setting of therapeutic decision-making. Indeed, the potential to integrate this biomarker data into treatment paradigms, while promising, is just starting to become explored.

**Table 1:** Subset analysis of prospective trials demonstrating improved prognosis with hpv-related oropharyngeal carcinoma.

Author	N	Dose	Induction	Concurrent	Outcomes
Fakhry (3)	96	70 Gy	Carbo/paclitaxel x2	Paclitaxel	86% vs 53%, 2yr PFS, p=0.02
Rischin (26)	172	70 Gy	None	CDDP +/- Tirapazamine	87% vs 72%, 2yr PFS, p=0.01 93% vs 86%, 2yr LRC, p=0.09
Ang (2)	323	70-72 Gy	None	Cisplatin	74% vs 43%, 3yr PFS, p<0.001 86% vs 65%, 3yr LRC, p<0.001
Lassen (4)	331	66-68 Gy	None	+/- Nimorazole	61% vs 35%, 5yr LRC, p<0.001
Lassen (4)	794	66-68 Gy	None	None	78% vs 64%, 5yr PFS, p=0.001 69% vs 57%, 5yr LRC, p=0.004
Worden (27)	66	70 Gy	Carbo/CDDP+5FU x1	Carbo/CDDP	85% vs 37%, 3yr PFS, p=0.001
Seiwert (28)	110	72 Gy	Carbo/paclitaxel/Cetux x2	Cetux/5-FU/hydroxyurea or Cetuximab/CDDP	84% vs 66%, 5yr PFS, p<0.01

Carbo, Carboplatin; CDDP, Cisplatin; 5FU, 5-fluorouracil; Cetux, Cetuximab; PFS, Progression-free survival; LRC, Local-regional control.

## Mechanisms of radio response

How HPV mediates radioresponse in the setting of squamous cell carcinoma of the head and neck is under active investigation and is likely related to a multitude of factors. The most direct explanation is that HPV infection and the subsequent molecular sequestration of the p53 and pRb proteins by the viral products E6 and E7 leads to a cascade of events including the interruption of cell cycle checkpoints and downregulation of cell cycle regulatory proteins culminating in increased genomic instability. As a result, the host tumor cell is left more susceptible to radiation-induced apoptosis. Both *in vitro* and *in vivo* studies, however, have demonstrated, that direct transfer of the E6/E7 genes or gene products into cells did not alter radiation resistance as would be expected (29). Pang et al, however, showed that transfection of the E6 transcript in HPV-negative squamous cell carcinoma cell lines resulted in sensitization to radiation-induced cell death (30).

The data on the interaction between HPV and DNA repair in mediating radiation sensitivity continues to prove provocative. Several studies have shown that the capacity of DNA repair might be hindered by HPV as measured by the persistence of double-strand breaks (31–33). Multiple mechanisms have been proposed as to how HPV might alter DNA repair capacities through homologous recombination and nonhomologous end-joining (34, 35). The role of altered DNA damage response is further supported by the observation that SMG-1, a key protein involved in DNA repair, was negatively correlated with HPV-positive oropharyngeal tumors (36). *In vitro*, decreased SMG-1 expression was seen in cell lines transfected with E6/E7 and such cells had enhanced radiosensitivity.

Other researchers have suggested that radiation has immunogenic properties itself and heightens the host immune response to viral antigens which are expressed on the cancer (37–39). How HPV recruits’ immune cells that potentiate the effects of radiation is under active investigation. Numerous studies have confirmed an immunologic mechanism to HPV-mediated radioresponse by demonstrating that the extent of tumor-infiltrating lymphocytes is associated with clinical outcome among patients treated for HPV-positive oropharynx cancer (39, 40). Indeed, the density and pattern of immune infiltrates in the tumor microenvironment is thought to be a byproduct of the HPV activation process in oncogenesis. Relatedly, the presence of regulatory T cells and PD-1(+) T cells and the levels of PD-1(+) cells were positively correlated with a favorable clinical outcome in HPV-positive compared to HPV-negative head and neck cancers (41). While speculative, this may reflect prior immune response in HPV-positive tumors, and radiation may possess a role in helping to re-activate this immune response. Indeed, the presence of tumor-infiltrating lymphocytes may itself be a prognostic marker of improved outcome, regardless of HPV-status (42, 43). The potential role of tumor-associated macrophages and regulatory T cells in mediating HPV-related radioresponse is also increasingly being investigated (38, 44, 45). These studies have demonstrated the importance of the microenvironment and its interaction with tumor cells in mediating radiation response in the setting of HPV-positive oropharyngeal carcinoma.

Regardless of the underlying mechanisms responsible for HPV-mediated radioresponse, laboratory work has confirmed the exquisite radiosensitivity of HPV-positive head and neck squamous cell carcinoma. Gupta et al. conducted a series of experiments using clonogenic survival assays of HPV-positive and HPV-negative cell-lines after exposure to various doses of radiation and showed that the former are characterized by markedly enhanced radiosensitivity (46). Similarly, Kimple et al. demonstrated that HPV-positive cell lines derived from squamous cell head and neck cancer exhibited greater intrinsic radiosensitivity characterized by prolonged G2-M cell-cycle arrest and increased apoptosis compared to HPV-negative cell lines (47). These findings were consistent with those of others showing that cell lines derived from HPV-positive oropharyngeal squamous cell were more frequently in G2 than those from HPV-negative tumors (48). In a series of experiments, Vlashi et al. showed that that the improved radiosensitivity of HPV-positive head and neck cancer might be due to the lower frequency of cancer stem cells and a decreased capacity to engage in radiation-induced dedifferentiation compared to HPV-negative head and neck cancer (49). While none of these studies have directly unraveled the secret of how HPV mediates an enhanced response to radiation, they have confirmed the observations from the clinic and have provided insights into how molecular biology can potentially be exploited to further treatment.

## Current treatment

Historically, the current standard for locally advanced HPV-positive and HPV-negative oropharyngeal cancer was identical– regardless of whether primary surgery or radiation therapy was the treatment upfront. As previously described, the HPV biomarker (via its surrogate p16) was not integrated into the staging system until 2016. Although this new system has been useful to categorize patients into varying prognosis based on standard treatment, how to utilize this information for clinical decision-making in the context of de-escalation is largely unknown. While it makes sense that patients with stage I and II (and even stage III) p16-positive oropharyngeal cancer might be the optimal candidates for de-escalation, this notion is speculative at present. Indeed, attempts to identify how treatment recommendations might differ from stage to stage have been hampered by the fact that many of the published studies on de-escalation have used the older staging system, which understandably makes extrapolations challenging (50). As a result, defining new standards of care by stage have remained elusive.

For patients opting for a non-surgical approach, the standard of 70 Gy with high-dose cisplatin chemotherapy has largely remained the same for decades. Alternative chemoradiation regimens which have been studied in the concurrent setting include weekly cisplatin or carboplatin, given alone or in combination with paclitaxel or 5-fluorouracil (51, 52). Concurrent weekly cetuximab with radiation and induction chemotherapy with multi-agent regimens such as taxotere, platinum and 5-fluorouracil followed by concurrent chemoradiation are additional treatment options that have been proposed (53–55). It is important to recognize that prospective trials designed to replace cisplatin with the targeted systemic agent, cetuximab, have shown that this approach may lead to inferior outcomes (56–58). The explanation for the lack of benefit associated with cetuximab might be because HPV-related tumors are less driven by underlying alterations in cell signaling pathways due to the oncogenic properties of HPV-oncoproteins E6 and E7. In other words, compared to HPV-negative carcinoma, HPV-positive oropharyngeal squamous cell carcinoma harbor mutational landscapes that are more devoid of driver mutations or alterations such as EGFR-overexpression (59). While the eligibility criteria varied between studies, they nonetheless have suggested that cisplatin should continue to be the standard when chemotherapy is utilized with radiation in the definitive treatment of HPV-positive oropharyngeal cancer. Although they do not truly address the question of which patients require chemotherapy for this disease, they nonetheless demonstrate the need for caution with ongoing attempts to pursue de-escalation. It must also be recognized that HPV confirmation was not standardly performed which raises the possibility that some patients with p16-positive disease actually did not have HPV-related disease. Additional studies analyzing whether immunotherapy can be utilized as an alternative are also ongoing (60–62). Although the side effect profiles of these various chemoradiotherapy regimens broadly differ, they generally are considered to be fairly intensive, particularly when combined with 70 Gy of radiation.

**Figure 2:**
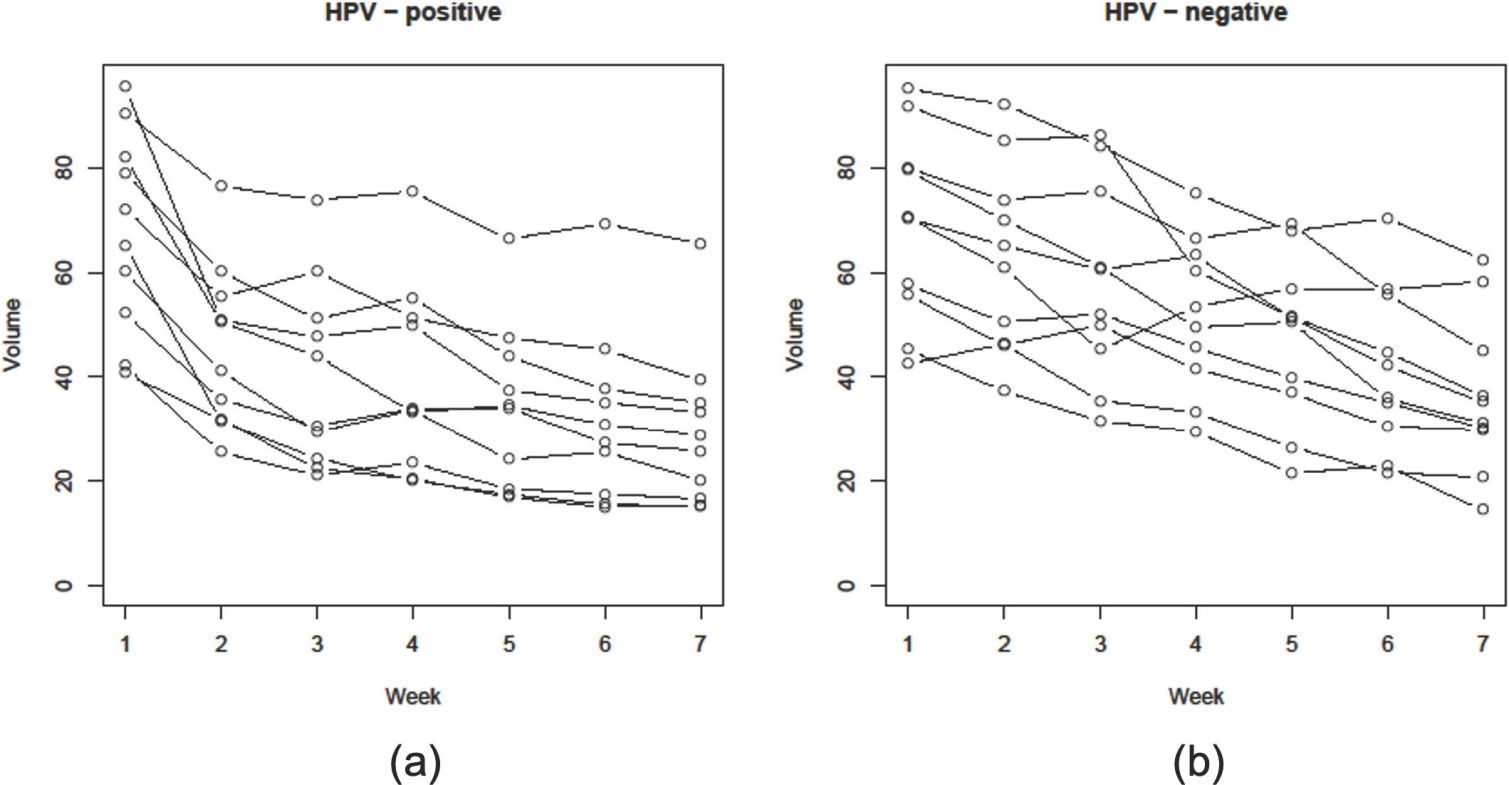
Graphical reduction in gross tumor volume (GTV) during a course of definitive radiation therapy for head and neck cancer among 10 patients each with **(A)** HPV-positive and **(B)** HPV-negative oropharyngeal squamous cell carcinomas whom were matched based on clinical and disease characteristics (38).

Given the provocative evidence attesting to the radiosensitivity of HPV-positive oropharyngeal cancer, an increased amount of attention has focused on ascertaining whether patients with locally advanced HPV-positive oropharyngeal cancer should be treated differently than those with HPV-negative tumors. Investigators from the University of California, Davis (**Figure 2**) using serial axial imaging to quantify tumor volume obtained longitudinally during the course of radiation to observe *in vivo* patterns of tumor response according to HPV status. This research showed that HPV-positive head and neck cancer tends to regress early during treatment, reaching a plateau by week 5-6, thus providing illustrative evidence that radiation doses can possible be reduced (63). In contrast, HPV-negative tumors were shown to respond relatively later during the course of radiation and more incompletely with respect to volume loss. The robust pattern of tumor reduction described for HPV-positive tumors was noted to be consistent with what was observed in the clinical setting.

The concept of de-escalation encompasses a variety of different strategies intended to make treatment gentler through a reduction in radiation, alteration in chemotherapy regimens, and/or elimination of either modality altogether. The overriding rationale for the interest in de-escalation stemmed from the ability to lessen the intensity of treatment while maintaining survival. However, how to best offer this approach to patients is uncertain, as various methods have been described; and the question of whether de-escalation is even ready for use outside of a clinical trial is hotly debated.

## Rationale for de-escalation

The historically observed rates of toxicity from head and neck irradiation are high. Given the understanding that the dose-limiting toxicity from chemoradiation has been related to effects on the mucosal and esophageal surfaces, reducing the radiation dose in selected patients with more favorable biology (e.g. HPV-positive tumors) has been proposed as an attractive option. Indeed, it has been well established that by effectively reducing radiation to the normal structures of the head and neck, there will be a consequent reduction in acute and late side effects—particularly related to swallowing—resulting in improved quality of life. Numerous prospective and retrospective data utilizing sophisticated probability models have demonstrated consistent dose-response relationships predicting toxicity for organs involved in salivary production, swallowing, and mucosal integrity (26–28, 64–67). For xerostomia, it has been long established that the ability to keep mean parotid dose below 26 Gy will significantly reduce the incidence of salivary dryness and preserve quality of life (66). Normal tissue complication probability models have observed that for every 1 Gy in mean dose, the likelihood of xerostomia increases by approximately 5% at 1 year after radiation therapy (67). An abundance of data has similarly shown that dose to anatomical structures thought to be responsible for swallowing is of critical importance in predicting acute and late toxicity from treatment. For instance, multiple studies have demonstrated that minimizing dose to the swallowing apparatus—the pharyngeal constrictor muscles, cervical esophagus, and cricopharyngeal inlet– may decrease the incidence of such side effect as dysphagia, esophageal stricture, trismus, and gastrostomy-tube dependence (26, 27, 64, 65). Between 55 Gy and 70 Gy, a strong linear relationship has been established linking dose to the inferior pharyngeal constrictor muscles and cricopharyngeal inlet with the late grade 3+ dysphagia as defined as gastrostomy-tube dependence (65). These data are consistent with published literature demonstrating that the threshold for radiation-induced long-term dysphagia likely exists at approximately 55 to 60 Gy, and dependent on dose-volume effects (26, 27). These same dosimetric variables have also been linked to complications such as aspiration pneumonia, severe dehydration, unintended weight loss, and malnutrition, as well as to psychosocial distress such as depression and anxiety (68–70). Even for peripheral neuropathy and osteoradionecrosis, presumably due to the development of fibrosis in the neck and/or as a direct effect of radiation-induced vasculitis, probability models have shown an increased likelihood of symptoms with doses exceeding 60 Gy (71, 72).

Given that the probability of developing most radiation-induced complications can be decreased by reducing the intensity and volume of radiation exposure, the potential of de-escalation to improve quality of life for patients undergoing treatment for head and neck cancer is profound. While the use of intensity-modulated and image-guided techniques to deliver radiation in a more customized fashion has become standard and has undoubtedly contributed to improvements in the therapeutic ratio, incidental exposure of radiation to anatomical structures that should be spared still inevitably occurs. This is because the location of many oropharyngeal tumors lie in such close proximity to these organs responsible for swallowing, speaking, and salivating, that it is nearly impossible to avoid subjecting them to radiation. By potentially decreasing toxicity without lowering cure rates, de-escalation of radiation dose for HPV positive tumors has the potential to improve therapeutic ratio by decreasing toxicity while maintaining high rates of disease control.

## Quality of life implications

It is increasingly recognized the limiting radiation dose to tissues such as the parotid gland, swallowing structures, larynx, and oral cavity, among others, has the potential to improve quality of life (73). As such, the goal of de-intensification is to improve quality of life while maintaining the excellent rates of cure observed in patients with HPV-positive oropharyngeal cancer. This effort is particularly relevant because traditional treatment using high-dose radiation frequently necessitates unintended breaks, hospitalization, and/or the use of intravenous hydration and enteral feeding. Indeed, it is well-established that concurrent chemoradiation for head and neck cancer has eclipsed the limits of acceptable long-term toxicity. In a combined analysis of late toxicity among patients treated on 3 chemoradiation prospective trials using cisplatin for head and neck cancer, Machtay et al. reported that nearly half of all patients experienced grade 3+ late toxicity related to laryngeal and/or esophageal dysfunction (74). Langendijk et al. similarly showed that the cumulative toxicity of radiation therapy has been shown to contribute to significant quality of life burden with respect to physical and psychosocial functioning (75). A longitudinal analysis by Chen et al. showed that although long-term function has seemingly improved among head and neck cancer patients treated over time due to advances in technology, a significant proportion of patients still rate their quality of life as poor at various points after radiation therapy (76). Consistent with the experiences of others, the less-than-optimal quality of life is related to toxicity largely with respect to swallowing and salivation. It is thus not surprising that the incidence of psychosocial distress has been shown to be high for patients after treatment, despite having long been cured of their disease. These studies, in aggregate, strongly suggest that traditional treatment using high-dose radiation (with or without chemotherapy) is associated with significant quality of life detriments which can unfortunately last a lifetime for patients.

## De-escalated radiation

Over the last decade, several prominent prospective trials have been published which have demonstrated promising outcomes with de-escalated radiation regimens using lower than conventionally accepted doses (**Table 2**). These have consistently shown that de-escalated radiation for HPV-positive oropharyngeal carcinoma can significantly decrease toxicity while maintaining the historically high rates of cure, thus largely validating the premise for which de-escalation was proposed (7–12). The popularity of this approach has been driven by the increasing recognition that HPV-related oropharyngeal cancer is exquisitely sensitive to radiation, as well as the increased desire of patients to avoid side effects.

**Table 2:** Prospective clinical trials on de-escalated radiation as initial treatment for hpv-positive oropharyngeal carcinoma.

First Author (Year)	N	Dose	Chemotherapy	PFS	OS	Time
Chen (2017) (7)	45	54-60 Gy	Induction Carboplatin/Paclitaxel Concurrent Paclitaxel	95%	98%	2-year
Chera (2019) (8)	114	60 Gy	Concurrent Cisplatin or None	86%	95%	2-year
Marur (2017) (10)	51	54 Gy	Induction Cisplatin/Paclitaxel/Cetuximab Concurrent Cisplatin	80%	94%	2-year
Misiukiewicz (2019) (11)	12	56 Gy	Induction Docetaxel/Cisplatin/5-FU Concurrent Carboplatin	83%	83%	3-year
Seiwert (2019) (9)	62	45-75 Gy	Induction Carboplatin/Paclitaxel	95%	98%	2-year
Yom (2021) (12)	150	60 Gy	None	88%	97%	2-year
Yom (2021) (12)	158	60 Gy	Concurrent Cisplatin	91%	97%	2-year

The evidence in favor of radiation alone for appropriately selected patients with HPV-positive oropharyngeal carcinoma is emerging. Based on historic data from the University of California, Davis and the Princess Margaret Hospital showing that radiation alone (to 70 Gy) is curative for patients with HPV-positive oropharyngeal carcinoma, investigators from Japan recently published a phase 2 trial showing 2-year progression-free survival (PFS) and overall survival (OS) of 94% and 100%, respectively (77–79). In a phase II study, investigators from the University of North Carolina reported on 114 patients who were treated with de-escalated radiation to 60 Gy (8). Notably, patients with higher tumor volume also received low-dose weekly cisplatin. With a median follow-up of 32 months, the 2-year PFS and OS was 86% and 95%, respectively. As importantly, the incidence of grade 3 or higher late toxicity was zero. Results from NRG HN-002, a phase II study of 306 patients randomized to de-escalated chemoradiation versus de-escalated radiation are particularly instructive. The investigators showed that de-escalated radiation alone to 60 Gy, as definitive upfront treatment, for locally advanced HPV-positive oropharyngeal squamous cell carcinoma, delivered using a 6 fraction per week regimen, achieved 2-year PFS and OS of 88% and 97%, respectively (12). Although the heterogenous nature of the subject populations precluded the drawing of definitive conclusions, these studies suggest that some patients with HPV-positive oropharyngeal squamous cell carcinoma can be treated with de-escalation and achieve excellent outcomes. Given the historically high rates of toxicity associated with chemotherapy, the use of radiation alone can be considered an attractive option for appropriate patients.

Indeed, the rationale for the elimination of chemotherapy is driven by the drive to decrease side effects and improve quality of life. The use of concurrent chemotherapy is well-known to dramatically decrease the tolerability of treatment compared to radiation alone (80, 81). In addition to its association with stand-alone side effects such as bone marrow suppression, renal failure, ototoxicity, and neuropathy, among others, the use of chemotherapy combined with radiation has been shown to exacerbate the effects of the latter (82). Studies have shown that the rates of hospitalization, treatment interruptions, and mortality are significantly higher among patients receiving concurrent chemoradiation compared to radiation alone (83, 84).

However, as previously discussed, findings from prospective studies showing that cetuximab is an inadequate substitute for cisplatin for HPV-positive oropharyngeal patients treated by chemoradiation must also be acknowledged. The results of RTOG 1016 conducted in North America randomized 849 patients with locally advanced p16-positive oropharyngeal cancer to radiation with high-dose cisplatin or weekly cetuximab showed inferior OS and PFS for the latter compared with the former (56). Eligibility included patients with T3-T4 tumors or N2a-N3 disease, as defined by the older 7^th^ edition staging system. The estimated 5-year OS was 78% in the cetuximab group versus 85% in the cisplatin group. Investigators from Europe published the “De-ESCALaTE” trial which randomized 334 patients with locally advanced p16-positive oropharyngeal cancer to radiation with high-dose cisplatin or weekly cetuximab (57). Notably, eligibility was defined using the older (7^th^ edition) staging system and included patients with T3-T4 or node-positive disease and minimal smoking history. While OS was not the primary outcome, the study showed a significant difference between cisplatin and cetuximab in 2-year OS (98% versus 89%) and 2-year recurrence (6% versus 16%) favoring cisplatin. The Trans-Tasman Radiation Oncology Group (TROG) randomized 189 patients from Australia and New Zealand to radiation with weekly cisplatin or weekly cetuximab (58). While there was no observed difference in the primary endpoint of symptom severity, the 3-year failure-free survival rates were 93% and 80%, respectively, among patients treated by cisplatin and cetuximab. Eligibility criteria included: AJCC 7th edition stage III (excluding T1-2N1) or stage IV (excluding T4 and/or N3 and/or N2b-c if smoking history >10 pack years and/or distant metastases) p16-positive. While these studies included a generally heterogenous group of patients, notably with respect to tumor volume, clinical stage, and smoking history, they suggest that not all patients might be appropriate for approaches de-intensifying treatment based on the alteration or elimination of radio-sensitizing chemotherapy. If anything, these studies point to a need for caution when designing de-escalation efforts moving forward.

The addition of chemotherapy (administered either before or with) de-escalated radiation is well-studied. Investigators from the University of California performed a multi-center, phase 2 trial, treating 45 patients with locally advanced HPV-positive oropharyngeal squamous cell carcinoma with 2 cycles of induction chemotherapy given 21 days apart, followed by de-escalated radiation to 54 Gy and 60 Gy to complete and partial responders (7). The PFS at 2 years was found to be 92%, and a significantly improved toxicity profile compared with historical regimens using standard radiation doses was observed. The gastrostomy-tube dependence rate at 6-months post-radiation and late dysphagia was zero. As importantly, prospective analysis of quality of life endpoints and pre- and post-therapy swallow studies showed that de-escalation dramatically improved function (85–87). For instance, patients treated by de-escalated radiation had decreased weight loss, depression, and opioid usage compared to contemporary control subjects who opted not to be treated with de-escalation.

The Optima trial was another phase 2 de-escalation study in which 62 patients with HPV-positive oropharyngeal squamous cell carcinoma were treated by induction chemotherapy with 3 cycles of carboplatin and nab-paclitaxel followed by de-escalated radiation (9). The 2-yr PFS was 94% for high-risk patients. The Quarterback trial was a randomized phase 3 study that directly compared reduced dose radiation to standard dose radiation after induction chemotherapy for locally advanced HPV-positive oropharyngeal cancer patients (11). After 3 cycles of docetaxel, cisplatin and 5-fluorouracil induction chemotherapy, patients with a clinical or radiographic complete/partial response were randomized to receive reduced (56 Gy) or standard (70 Gy) dose radiation with weekly carboplatin. Among the 20 patients randomized, the 3-year PFS rates were not significantly different at 88% and 83% for those receiving standard and reduced dose radiation, respectively. Lastly, NRG HN02 showed excellent rates of survival with concurrent chemoradiation to 60 Gy (12). Notably, in this trial the addition of concurrent cisplatin to de-escalated radiation reduced the 2-year local failure rate from 9% to 3% although it was unclear which subset of patients benefited the most. When the 2-year PFS and OS rates were analyzed, no differences were observed between patients treated by de-escalated radiation with or without chemotherapy.

Lastly, minimally-invasive operative techniques using transoral robotic surgery (TORS) has also been proposed as a means of de-escalating treatment for HPV-positive oropharyngeal cancer (88–90). As an initial treatment, TORS has been shown to be effective in resecting the primary cancer with minimal morbidity. Additional data from the University of Pennsylvania group has further suggested that eliminating postoperative radiotherapy to the primary site for selected patients with oropharyngeal cancer treated by TORS results in high local control and optimal function (91, 92). Another published study from Washington University has suggested that adjuvant chemotherapy may not be necessary for any patients with HPV-related oropharynx cancer, even in the setting of risk factors typically prompting its use such as extracapsular disease spread (93). Enthusiasm for the use of TORS, however, may have been dampened by the results of the ORATOR trial which randomized patients with newly diagnosed HPV-positive oropharyngeal cancer to either initial TORS or to primary radiation (94). While OS and PFS were the same between the 2 arms, patients randomized to TORS had decreased swallowing function at 1-year, which translated into inferior quality of life. Notably, a subsequent randomized trial comparing initial TORS to primary radiation using de-escalated doses was conducted by the same investigators and was halted prematurely due to excessively high grade 5 toxicity in the TORS arm (95). Nonetheless, prospective studies published by the Eastern Cooperative Oncology Group (ECOG) and the Mayo Clinic have shown that reduced doses of radiation (to 30 to 50 Gy) in the post-operative setting may be reasonably delivered after TORS (96, 97).

These prospective trials, in aggregate, have established de-escalated radiation as a feasible treatment option for patients with HPV-positive oropharyngeal cancer. Not only do they demonstrate that de-escalated radiation achieves exceptionally encouraging rates of PFS and OS, but they strongly suggest that de-escalation was associated with meaningful improvements in quality of life and functional outcomes. While preliminary, these data effectively validate the premise underlying de-escalation and provide encouraging evidence that this strategy will be adopted in a more widespread fashion in the future. Notably, a post-hoc analysis of perspectives and attitudes of subjects treated on the University of California de-escalation trial showed that nearly all patients were satisfied with their decision and any regret was nearly non-existent (87). Further evidence supporting de-escalation was provided by Gabani et al. who analyzed data from the National Cancer Database to identify 759 patients with HPV-positive oropharyngeal cancer who were treated with definitive radiation with or without chemotherapy (98). Using a propensity score model to minimize imbalances between arms, the investigators showed no differences in outcome between patients treated to 66 Gy or higher and those treated to lower doses. Furthermore, no benefit to concurrent chemotherapy was observed. Yang et al. similarly conducted a meta-analysis of 13 studies for patients with HPV-positive oropharyngeral cancer and concluded that the 2- and 3-year OS rates in the de-escalated radiation group (96% and 92%, respectively) were superior to those in the standard-dose group (88% and 87%, respectively) leading them to conclude that alleviates the treatment toxicities without compromising survival in this population (99).

## De-escalation: next steps

Continued progress to better refine selection criteria as well as to dynamically monitor treatment response will define the evolution of de-escalation for HPV-positive oropharyngeal cancer. At present, the only clinical–pathologic factor (other than AJCC cancer stage) that is used for risk stratification is smoking history. Future advances in de-escalation will need to incorporate a combination of clinical, radiological, and biological data—helping to apply principles of precision medicine to this approach.

The use of cell-free DNA to quantify disease burden and to longitudinally monitor response has been proposed to further individualize care as numerous studies have prospectively demonstrated its utility for prognostication and surveillance purposes for patients treated by radiation for HPV-related oropharyngeal cancer (100–102). The incorporation of other immunologic biomarkers such as PD-1/PDL-1 in conjunction with HPV has also been studied as a more powerful means to refine risk stratification (103, 104). Corredor et al. recently employed image processing and machine learning to develop an imaging biomarker that quantitatively characterized the spatial patterns of tumor-infiltrating lymphocytes and surrounding nucleated cells in digitized hematoxylin and eosin slides of HPV-positive oropharyngeal cancer patients (105). The investigators then showed how this model could be implemented in current staging systems to refine prognostication and to aid in the selection of patients potentially for de-escalation. The utility of pre-treatment circulating leukocytes as a predictive measure of radiation response has also been proposed (106, 107). Unraveling the mechanisms of radiosensitivity may further lead to the development of therapeutic cancer vaccines, which are now being studied (108, 109). The potential of high-yield, next generation sequencing panels to cluster tumors into even more distinct subtypes based on immunogenomics has also been described (110, 111). Others have suggested that expression of cancer stem cell markers in HPV-positive oropharyngeal squamous may help further characterize biological behavior and identify patients who derive the most benefit from de-escalation (112). Indeed, attempts to discern gene profile signatures that might be useful for risk stratification continue to be explored (113).

The explosion of radiomic information also has the potential to identify who may or may not be eligible for de-escalation, both at diagnosis and midway through radiation. For instance, investigators from China used a radiomics signature of intra-tumoral and peri-tumoral regions to predict which patients might benefit from the addition of chemotherapy to radiation for HPV-related oropharyngeal cancer (114). Another study showed that radiomics can outperform traditionally used clinical factors to characterize HPV-related oropharyngeal squamous cell carcinoma (115). The potential of multi-parametric magnetic resonance imaging (MRI) including diffusion-weighted sequences is also starting to become recognized and may be incorporated into risk stratification schemes in the future (116, 117). Other investigators have suggested that hypoxia monitoring using novel radiotracers can be useful for discerning the most optimal patients for de-escalation (118). In a prospective study utilizing fluoromisonidazole-positron emission tomography (F-MISO-PET) to image hypoxia during radiation, they showed that radical reduction in radiation dose to 30 Gy for those with no pretreatment hypoxia or in whom hypoxia had resolved within the first 2 weeks of initiating radiation might be feasible (119). All in all, tremendous resources are being invested in the identification and development of phenotypic signatures which might predict treatment success for patients opting for de-escalation. The use of machine learning and artificial intelligence is now being investigated as a means to make this process more efficient and clinically practical (120–122).

## Conclusion

Given its demonstrated ability to dramatically preserve quality of life and functioning while maintaining high rates of cure, de-escalated radiation has emerged as an attractive option in the management of HPV-positive oropharyngeal cancer. This strategy is seemingly well-supported by the depth and breadth of data that has been published reporting on outcomes of de-escalated radiation for HPV-positive oropharyngeal cancer. Indeed, the reality of clinical decision-making for HPV-positive oropharyngeal cancer has evolved to the point where patients are now routinely demanding de-escalated radiation. Findings from a recent patterns of care analysis demonstrated that de-escalated radiation has become increasingly offered to patients with HPV-positive oropharyngeal cancer as standard treatment (123). Given the preliminary nature of the data to date and the failures of prospective trials attempting to de-escalate treatment with cetuximab, caution must be exercised. However, the fact that patients are demanding and being offered de-escalation outside of clinical trials naturally raises the question of whether a prospective clinical study randomizing subjects to de-escalation versus standard high-dose radiation could ever successfully be performed given that patients, many of whom are educated, are increasingly preferring the former (124).

While is now obvious that HPV-positive and HPV-negative oropharyngeal cancer represent distinct entitle with differing prognosis, the therapeutic implications remain unclear (125–127). While data has steadily emerged, that treatment should be individualized for the subgroup of patients with HPV-related oropharyngeal cancer, exactly how to do so remains uncertain (128). While patients with low-risk disease (low volume and minimal smoking history) can likely be effectively treated with de-escalated radiation alone, those with higher-risk disease (bulky, high-volume disease and/or patients with significant smoking histories), appear to benefit from the addition of chemotherapy to de-escalated radiation. However, these paradigms continue to evolve as studies contribute to an improved understanding of HPV-related oropharyngeal cancer leading to refinement in risk stratification schemes. While enthusiasts argue that the data robustly supports the integration of de-escalation into contemporary practice; skeptics point out that the published data is still relatively preliminary and makes it difficult to make definitive recommendations. Based on the emerging evidence, as well as on the explosion in interest from patients and physicians alike, well-designed clinical trials are urgently needed to better refine selection criteria for de-escalation and to stratify patients with newly diagnosed oropharyngeal cancer into the appropriate means of treatment.

## Author contributions

The author confirms being the sole contributor of this work and has approved it for publication.
